# Vertical and horizontal biogeographic patterns and major factors affecting bacterial communities in the open South China Sea

**DOI:** 10.1038/s41598-018-27191-w

**Published:** 2018-06-11

**Authors:** Yi Li, Lin-Lin Sun, Mei-Ling Sun, Hai-Nan Su, Xi-Ying Zhang, Bin-Bin Xie, Xiu-Lan Chen, Yu-Zhong Zhang, Qi-Long Qin

**Affiliations:** 1 Marine Biotechnology Research Center, State Key Laboratory of Microbial Technology, College of life science, Shandong University, Jinan, 250100 China; 2 Laboratory for Marine Biology and Biotechnology, Qingdao National Laboratory for Marine Science and Technology, Qingdao, 266237 China

## Abstract

Microorganisms display diverse biogeographic patterns in the three-dimensional contiguous seawater. The distance-decay relationship, the change in species composition similarity between different communities over a geographic distance, is a commonly observed biogeographic pattern. To study biogeographic patterns and the corresponding driving forces, the bacterial distance-decay patterns along the horizontal and vertical dimensions in the South China Sea (SCS) were investigated through the sequencing of partial 16 S rRNA gene regions. Along the horizontal geographical distances (up to ~1000 km), no significant distance-decay pattern in community compositions was observed in any of the tested seawater layers. However, vertical depths (up to ~4 km) had strong effects on bacterial community variation, which was apparently governed by dispersal barriers due to limited water mass mixing. In addition, community variations in the vertical direction were strongly correlated with the prominent variation of environmental factors. Apparently, the changes in bacterial community compositions along vertical distances were much greater than those along horizontal distances. The results showed that the distance-decay relationship in bacterial communities at the medium spatial scale was associated with vertical depth rather than with horizontal distance, even though the horizontal distance is much larger than the vertical distance in the open SCS.

## Introduction

The ocean is the largest three-dimensional contiguous aquatic environment on the Earth and contains approximately 10^6^−10^9^ microbial taxa and 3 × 10^28^ microbial cells^1,2^. These marine microorganisms play vital roles in diverse oceanic biogeochemical processes and are recognized as indispensable agents of nutrient cycling and climate change on a global scale^3,4^. Hence, understanding the patterns of marine microbial distribution and the factors that drive these distribution patterns is essential for predicting the response of marine microbial ecosystems to future environmental changes^2,5^. In recent years, there has been extensive research regarding microbial biogeography, which studies the diversity of microorganisms over space and time^6,7^. Due to their small sizes, high dispersal abilities and large population sizes, microorganisms are thought to be globally distributed and primarily selected by environmental factors^8^. This situation is very suitable for marine microorganisms because global seawater is a dynamic and contiguous system and horizontal and vertical currents can disperse marine microorganisms to every corner of the world’s oceans, as demonstrated in the Southern Ocean^9^ and in deep seas^10^. In addition to the dispersal rate being governed by ocean currents, microbial biogeographic patterns are also dependent on selective processes^6^, including environmental pressures (e.g., wind, sunlight and nutrient availability)^11,12^ and biotic interactions (e.g., algal blooms, viral lysis and grazing)^13,14^. Additionally, marine microbial communities follow scale-dependent spatial biogeographic patterns^2,5^. However, contradictory biogeographic patterns have been observed in marine microorganisms. Some studies, such as the microbial seed bank theory^15^, have shown the cosmopolitanism of marine microbial species characterized by the lack of biogeographic patterns. Meanwhile, other studies have shown that marine microbial species are provincial, i.e., different environments exhibit different microbial abundances and diversities^16^. Several factors can lead to contradictory biogeographic patterns, including differences in spatial scales, taxonomic resolutions, sequencing platforms and regions, as well as the habitat types considered for the studies of microbial biogeographic patterns.

A commonly studied microbial biogeographic pattern is the distance-decay relationship, which describes how similarities in community compositions vary with geographical distance^2,14,17^. The ocean is a three-dimensional contiguous aquatic system where microbial diversity can be affected by horizontal and vertical distances. There are sinking and upwelling currents that can vertically mix seawater and microorganisms. Horizontal currents can also mix the seawater at a single depth. Therefore, there are distance-decay relationships along the vertical depth and horizontal distance in seawater. Because of the stratification of the ocean, distance-decay relationships are prominent along the vertical depth^18^. However, it remains unclear if there are any differences between the patterns of distance-decay relationships along the vertical depth and horizontal distance, and if there are differences, it is unclear which factors drive these differences.

Located in the subtropical and tropical areas adjacent to the western North Pacific Ocean, the South China Sea (SCS) is the world’s largest marginal sea, with a maximum depth of over 5000 m^4^. The water of the SCS has frequent and complicated horizontal and vertical currents^19^. The surface ocean currents are mainly driven by monsoons and changes in seasons. Winter currents flow northeast to southwest, but summer currents flow in the opposite direction. The upper layer (0–200 m) of seawater circulates cyclonically in winter but anticyclonically in summer^20^; this is also true of the circulation in the bottom layer of the SCS shelf ^21^. Meanwhile, large-scale horizontal flow is observed in the intermediate current of the SCS^19^. The Kuroshio branch intrudes into the SCS through the Luzon Strait in winter, but sometimes this intrusion occurs in summer, which greatly affects hydrological states and circulation structures^22,23^. With low temperature and high salinity, the Pacific intermediate waters near the east side of the Luzon Strait are characterized by high density^24^. The Pacific waters sink when they enter the SCS, and the local deep-water masses of the SCS upwell to compensate for the sinking movement^25^. Besides, monsoon winds cause offshore delivery of seawater and lead to the formation of upwelling and sinking currents^20^. In addition, submarine mountains can regulate vertical currents^10,19^. All these currents constantly mix the seawater in vertical and horizontal directions.

**Figure 1:**
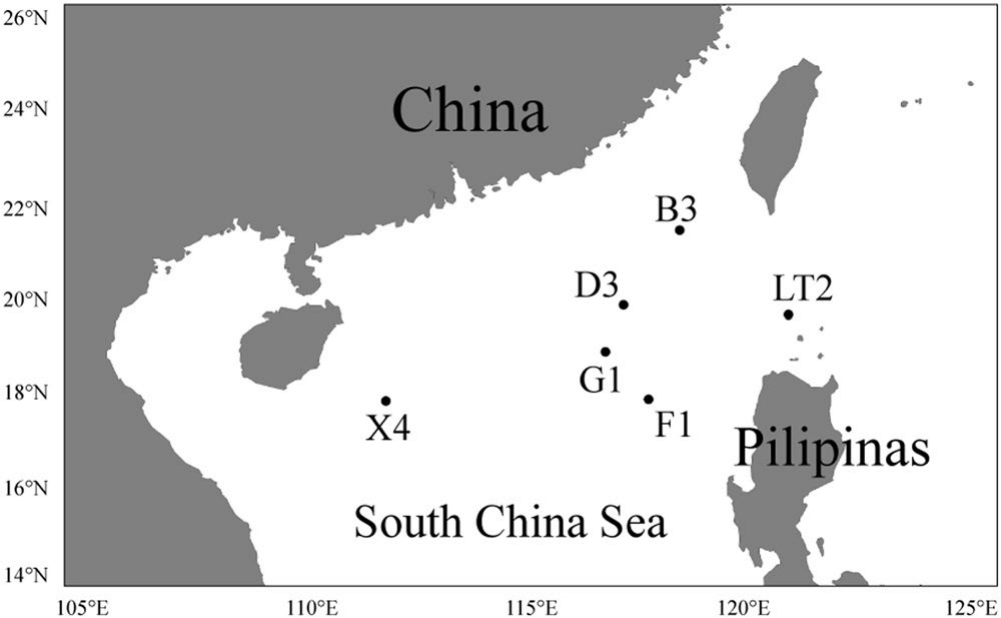
Map locations of the sampling sites in the open South China Sea.

Therefore, the SCS environment is an ideal region for the study of the biogeographic patterns of marine bacterial communities along the horizontal and vertical directions. In this study, bacterial communities from different samplings at horizontal distances up to ~ 1000 km and vertical distances up to ~ 4 km were systematically investigated to study and compare the distance-decay relationship patterns along horizontal and vertical distances (Fig. 1). Furthermore, the major factors driving the biogeographic patterns were also explored.

## Results and Discussion

### Bacterial taxonomic assignment and community variance

After quality control, a total of 3,128,028 high-quality sequences were retained for post-run analysis. A total of 2665 OTUs were assigned at a 3% dissimilarity threshold. The number of OTUs changed from 373 to 1310 across 24 samples, with the highest OTU numbers seen in the 200-m layer (Wilcoxon test, *P* < 0.05) (Supplementary Table S1). For Shannon diversity, no remarkable difference was observed between samples collected from the same layer at two different sites. However, there were clear differences in community diversities between the various vertical layers (Supplementary Fig. S1). The Shannon index of the surface water was dramatically higher than that of deep water (800-m, *P* = 0.026; Deep, *P* = 0.008) and was comparable to that of the 200-m layer.

In total, 35 bacterial phyla were found in all the samples. Proteobacteria had the largest proportion in most of the upper seawater samples. Firmicutes were abundant in the intermediate and deep layers. Actinobacteria, Cyanobacteria, Bacteroidetes and Acidobacteria were also present at a high abundance across all samples (Supplementary Fig. S2). The microbial phyla network, with 21 nodes and 72 edges, can be divided into two community groups. The degrees of Acetothermia, Acidobacteria, Thermodesulfobacteria and Gemmatimonadetes were 10, which indicated a high incidence of inter-phylum correlations (Supplementary Fig. S3a).

**Figure 2:**
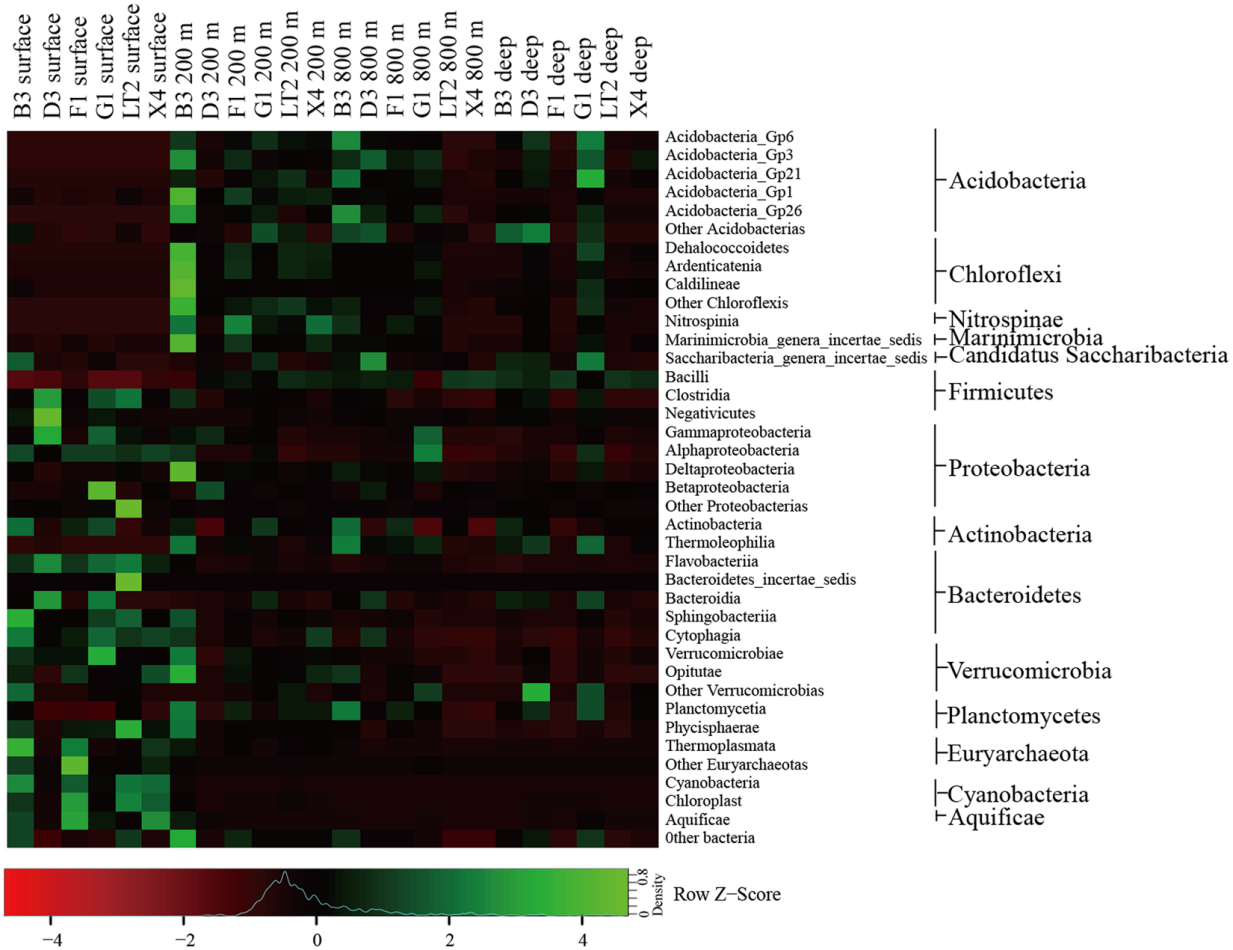
Heatmap showing the phylogenetic abundances of major bacterial clades at the class level across all the samples. Color bars represent the row-scaled value, in which a blue curve illustrates the distribution density percentage.

To identify phylogenetic differences at finer taxonomic levels, bacterial abundance variations between different layers were analyzed at the class level (Fig. 2). As the most abundant group in Proteobacteria, Gammaproteobacteria showed a decreasing trend in community abundances along the vertical depth, with the abundance changing from 29.3% in the surface zones to 13.2% in the Deep areas. This trend was also observed in Alphaproteobacteria. The distribution pattern was in agreement with previous reports that Cyanobacteria and the classes α- and γ-proteobacteria were the predominant surface-seawater bacterial groups in the SCS^4^. Burkholderiales and Rhodocyclales, with high abundance in Betaproteobacteria, were observed in our sampling sites, though they were predominant in freshwater areas instead of saltwater areas^26^. Cyanobacteria accounted for 23.7% of the bacterial communities from surface sites, and a majority of them belonged to the GpIIa family, including *Prochlorococcus* and *Synechococcus*. Furthermore, *Prochlorococcus* was observed in the deep seawaters, as reported previously^1^. Deep-sea *Prochlorococcus* may have evolved particular characteristics, such as the ability to take up and use dissolved organic carbon, to survive in the aphotic zone^27^. The class Flavobacteriia (predominantly composed of Flammeovirgaceae) had high abundance in the surface layer likely due to its important functions. The genus *Zunongwangia*, which is affiliated with Flammeovirgaceae, has the important ability to hydrolyze carbohydrates and proteinaceous organic nitrogen-containing compounds^28^. Genomic analyses of *Polaribacter* revealed a key function of this genus in the degradation of sulfated polysaccharides that are major components of algal cell walls^29^. Previous reports have demonstrated that Flavobacteriia can mediate algal organic matter degradation, supporting the growth of the SAR11 clade and marine Gammaproteobacteria^12,30^. This phenomenon was consistent with our network analysis at the family level, which showed that Flammeovirgaceae had wider positive correlations with members from other phyla, including Alphaproteobacteria, Gammaproteobacteria and Cyanobacteria (Supplementary Fig. S3b). Thus, some synergistic heterotrophic microorganisms in marine waters might grow partly due to Flavobacteriia-derived organic products.

Generally, the observed bacterial community compositions and variation trends in this study are consistent with previous studies in this area.

### Bacterial distance-decay pattern along the horizontal distance and vertical depth

**Figure 3:**
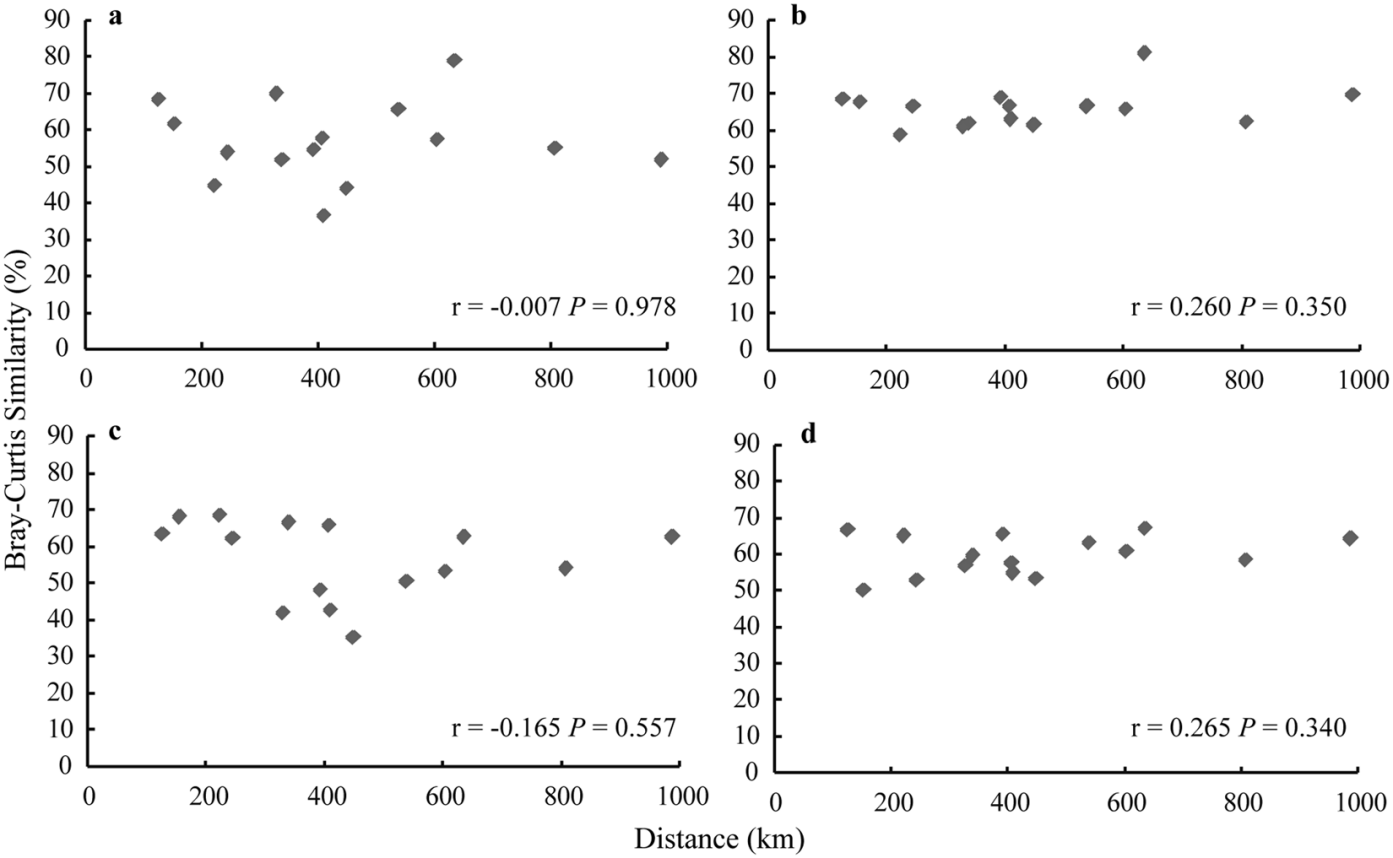
Correlations between bacterial community similarities at the same depth versus geographical distance. Bacterial communities were structured on Bray-Curtis similarity. Correlations from (**a**) Surface, (**b**) 200-m, (**c**) 800-m and (**d**) Deep layers are represented as Pearson correlations.

Based on an average Bray-Curtis similarity and Jaccard similarity between each pair of samples, the bacterial communities from the four seawater layers were not significantly correlated with horizontal geographical distance (126 km to 989 km) (by Pearson correlations (*P* > 0.05)) (Fig. 3 and Supplementary Fig. S4). This result was inconsistent with a previous study in the SCS that showed a biogeographic distribution of bacteria due to environmental control and dispersal limitation^31^. It was reported that distance-decay patterns of beta-diversity (variations in community composition) are dependent on spatial scales^5^. Therefore, the medium scale of 100–1000 km between the sample sites in the open SCS may be a limiting factor, leading to the absence of a distance-decay pattern. Notably, the environmental parameters (nitrate, nitrite, DOC, pH, phosphate and temperature) versus the geographic distance in each layer showed no remarkable difference (Wilcoxon test, *P* > 0.05) (Table 1). This showed that environmental perturbation did not drive changes in microbial communities across horizontal distances. With native SCS water masses exchanged at each water layer, there was no obvious dispersal limitation leading to reduced differences in bacterial communities^19^. In addition, the Kuroshio branch intruded into the north of the SCS through the Luzon Strait with large-scale surface and subsurface circulations; these horizontal current flows also decreased bacterial dispersal limitation. Average Bray-Curtis similarities of bacterial communities from Surface, 200-m, 800-m and Deep layers were 56.8%, 66.0%, 56.4% and 59.7%, respectively (Supplementary Fig. S5a). The trend was similar with Jaccard similarities (Supplementary Fig. S5b). These relatively high community similarities in the same water layers implied a weak distance-decay pattern along the horizontal direction.

**Table 1:** Environmental variables of all the seawater samplings.

Station	Depth (m)	Temperature (°C)	Salinity (PSU)	NO_2_^−^ and NO_3_^−^ (μmol L^−1^)	PO_4_^3−^ (μmol L^−1^)	DIC (μmol kg^−1^)	DO (μmol kg^−1^)	pH	DOC (μmol kg^−1^)
B3 surface	7	30.341	33.887	n.d.	n.d.	n.a.	n.a.	8.1	71.7
B3 200 m	201	14.910	34.552	15.1	1.1	2114.7	145.5	7.8	50.7
B3 800 m	801	5.404	34.477	35.3	2.5	2289.7	94.1	7.5	43.3
B3 deep	2500	2.314	34.618	38.4	2.8	2333.7	119.5	7.5	40.3
D3 surface	5	29.380	33.949	n.d.	n.d.	1927.7	197.7	8.1	72.0
D3 200 m	199	14.545	34.528	15.1	1.0	2117.3	149.1	7.8	51.7
D3 800 m	793	5.062	34.495	n.a.	n.a.	n.a.	n.a.	n.a.	43.4
D3 deep	2078	2.397	34.614	38.4	2.8	2335.5	108.7	7.5	41.4
F1 surface	5	29.373	33.327	n.d.	n.d.	1894.7	198.6	8.1	67.0
F1 200 m	198	15.041	34.526	17.7	1.3	2124.3	118.2	7.8	52.9
F1 800 m	793	6.084	34.460	34.9	2.6	2278.9	83.0	7.5	44.9
F1 deep	3795	2.363	34.624	38.4	2.8	2336.3	114.7	7.5	39.7
G1 surface	5	29.419	33.684	n.d.	n.d.	1910.4	196.4	8.1	73.9
G1 200 m	199	14.585	34.533	15.4	1.1	2116.1	143.5	7.8	51.6
G1 800 m	793	5.789	34.469	35.0	2.6	2278.7	85.1	7.5	42.5
G1 deep	2472	2.379	34.619	38.4	2.8	2338.2	108.3	7.5	41.3
LT2 surface	7	28.843	34.633	0.1	n.d.	1947.1	205.9	8.1	70.6
LT2 200 m	200	15.829	34.610	10.6	0.8	2082.8	173.3	7.9	50.0
LT2 800 m	801	5.068	34.421	36.4	2.7	2285.2	91.7	7.5	42.9
LT2 deep	2500	2.234	34.616	38.9	2.9	2333.1	118.8	7.5	n.a.
X4 surface	5	30.389	33.442	n.d.	n.d.	1899.2	195.5	8.1	73.4
X4 200 m	199	15.587	34.539	15.4	1.1	2114.3	136.4	7.8	52.4
X4 800 m	794	5.307	34.492	34.3	2.5	2283.9	85.8	7.5	44.7
X4 deep	2392	2.333	34.621	38.4	2.8	2342.4	109.0	7.5	39.8

n.a., no analysis; n.d., non detectable.

**Figure 4:**
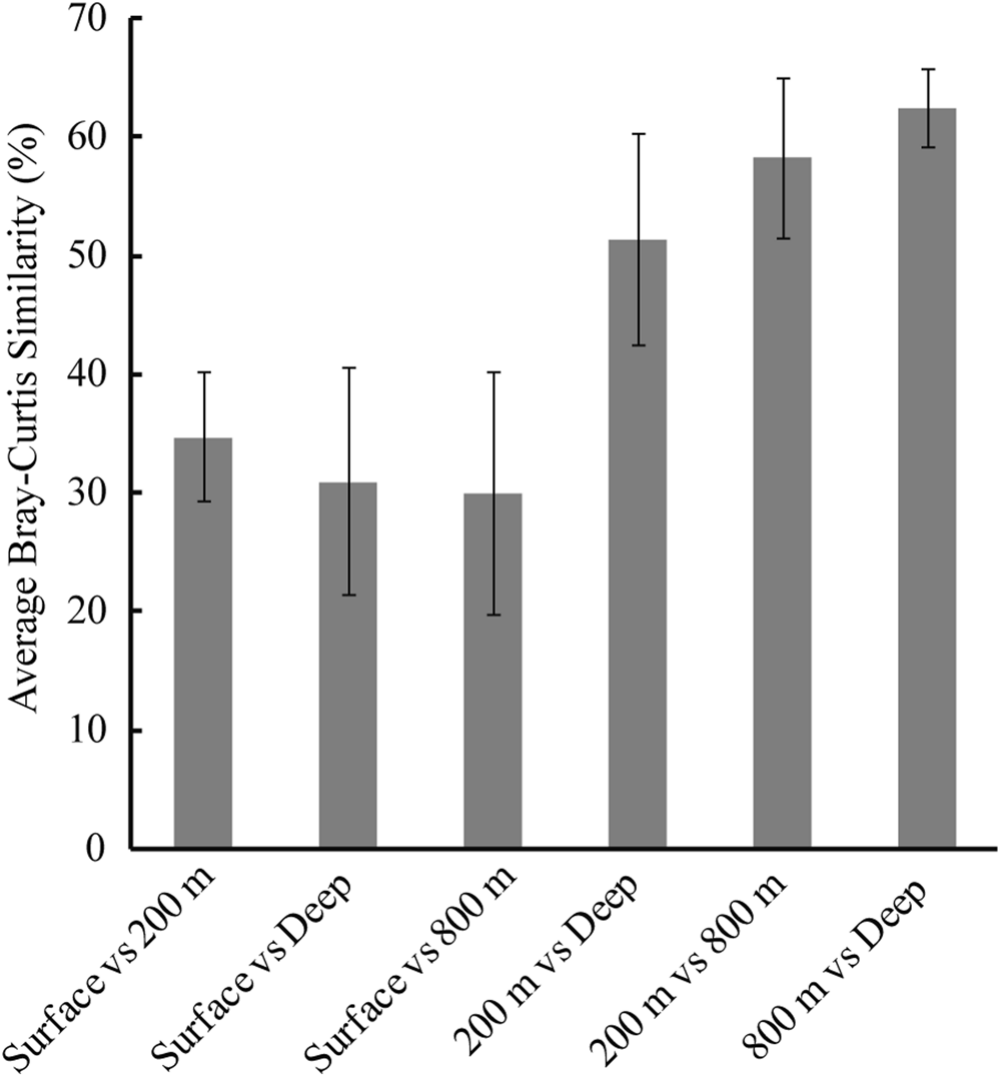
Comparison of bacterial community similarities at different depths. The average Bray-Curtis similarity value was calculated based on the similarity values between each pair of samples from different depths.

**Figure 5:**
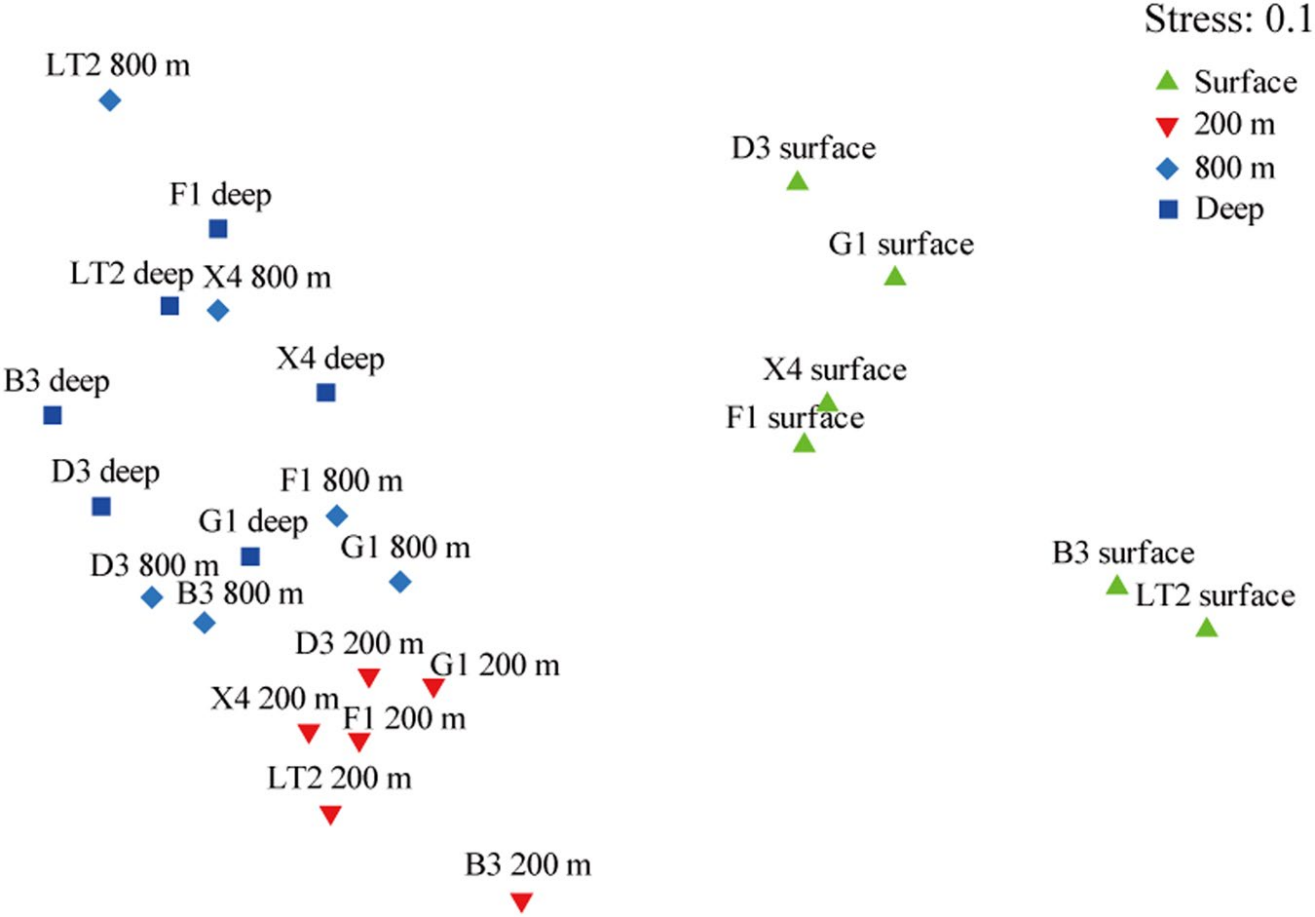
Nonmetric multidimensional scaling (NMDS) ordination of bacterial communities. The ordination was built based on the rank order of bacterial Bray-Curtis similarity. The four colored groups represent the four seawater layers: a surface group, a 200-m group and an 800-m-deep mixed group.

**Figure 6:**
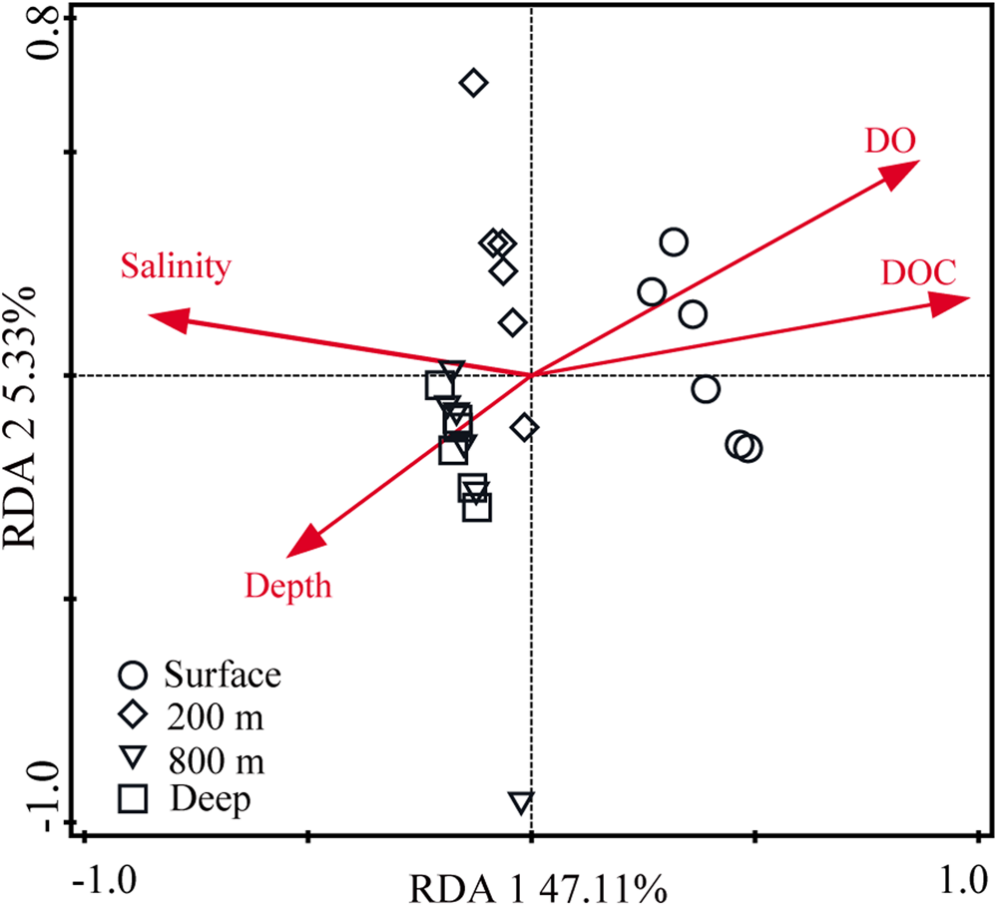
Redundancy analysis demonstrating the relationship between environmental variables and seawater samples. The environmental factors and various sampling depths are indicated by red arrows and different shapes, respectively.

The pronounced ocean stratification implied that vertical depth would have a strong effect on microbial community diversity and result in an obvious distance-decay relationship. Previous studies had reported a universal biogeographic pattern of variations in microbial community similarity versus geographic distance^2,14,17^. In this study, significant bacterial community differences between different water layers were observed along the vertical distance (0.2 km to 3.8 km), which was far shorter than the horizontal distances measured (>100 km) (Fig. 4). Besides, the average Bray-Curtis similarities of bacterial community between the surface-layer zones and the 200-m, 800-m and Deep layers were 34.8%, 30.0% and 31.0%, respectively. These low similarities that also were supported by Jaccard similarity indice hinted at obvious variations in community structures between the surface and other layers (Supplementary Figs S6 and S7). The unconstrained non-metric MDS analysis clearly showed that surface seawater and the other three water layer samples had different bacterial compositions that clustered into two larger groups (Fig. 5). This distribution pattern was in agreement with the constrained RDA result, which showed that the two larger groups were remarkably divided by the RDA 1 axis and that the intra-group members changed along the RDA 2 axis. Accordingly, a remarkable separation between the surface seawater group and the groups from the other layers was confirmed (ANOSIM, Surface vs 200 m, R = 0.98, *P* = 0.002; Surface vs 800 m, R = 0.97, *P* = 0.002; Surface vs Deep, R = 0.99, *P* = 0.002). Furthermore, the heterogeneity observed in the surface seawater community structures was relatively higher than that observed in the intermediate and deep layers. This high bacterial heterogeneity of surface seawater may be due to sunlight and more active conditions at the surface, as reported in other studies (Figs 5 and 6)^32^.

The bacterial communities of the 200-m layer were also significantly different from those of the 800-m (ANOSIM, R = 0.53, *P* = 0.002) and Deep (R = 0.92, *P* = 0.002) layers. However, the bacterial communities of the 800-m and Deep layers had a high similarity value of 62.6% and barely showed a measurable difference (ANOSIM, R = 0.14, *P* = 0.134). This result showed that the bacterial community compositions changed drastically from the surface to the 800-m layer and then changed slowly as the depth increased. Thus, vertical bacterial distance-decay patterns in the seawater system showed a stepwise change instead of a smooth gradient. Meanwhile, no clear difference in bacterial communities was found within the medium-scale horizontal distances sampled.

### Environmental factors driving the bacterial distance-decay pattern

It has been reported that most bacteria and archaea are limited to unique habits, mainly due to different environmental driving forces^33,34^. In this study, the environmental factors at each water layer were not significantly correlated with horizontal geographic distance, and no co-variance was found (Mantel test, *P* > 0.05). However, significant differences of environmental variables were observed along the vertical depths. The temperature of all samples, ranging from 2 to 30 °C, showed an obvious increasing trend from deep regions to surface layers; a similar trend was seen for the levels of the organic nutrient DOC. In contrast, the levels of the inorganic nutrients measured, namely, PO_4_^3−^, NO_2_^−^, NO^3−^ and DIC, were significantly lower in surface layers than in deep waters. The pH value was significantly higher in the upper zones than in the deep regions (Wilcoxon test, *P* < 0.05), but there was no pH difference between the 800-m layer and the Deep layer (Table 1).

To find the determinant environmental factor influencing bacterial composition patterns, RDA was performed across all the samples. The synergetic driving force of all the environmental variables provided a high explanatory power (approximately 77%) for the change of bacterial assemblages, suggesting effective impacts on variability in community patterns across seawater depths. After the removal of colinearity effects with Monte Carlo permutation tests, the four environmental variables were seen to significantly contribute to the relationships between major community distributions and the corresponding environmental factors (*P* < 0.05). The DOC content had the highest RDA explanatory power (44.6%) on the main phyla from different depths, followed by DO (F = 10.7, *P* = 0.002), salinity (F = 10.2, *P* = 0.002) and depth (F = 3.4, *P* = 0.002). The variable that best correlated with microbial community structures was DOC (r = 0.581, *P* < 0.001) and the top pair of environmental factors was DOC and salinity (r = 0.583, *P* < 0.001), as determined by BIOENV and BVSTEP analyses. A large DOC pool was reported to be present in the ocean, and the available compounds from this pool shape microbial community composition^35,36^. Therefore, it is reasonable that DOC content was a determinant environmental factor affecting bacterial community structures and their vertical distance-decay patterns in our study. Furthermore, clear differences in all the tested environment variables between different layers (except for salinity between surface zones and other layers, and DO and pH between the 800-m and deep layers) implied the formation of specific habitats, facilitating the vertical variations of bacterial communities (Wilcoxon test, *P* < 0.05). However, for the same horizontal seawater layer, the environmental variables were not significantly different between any two sites. The environmental factor analyses can partially explain why bacterial communities showed obvious discrepancies along vertical depths but no prominent community variance along the horizontal distances.

### Effect of currents on bacterial biogeographic patterns

In addition to the major influence of environment variables on the variations of microbial community compositions, ocean currents also may affect microbial biogeographic patterns^4,20,37^. It was reported that water masses in open areas of the SCS were divided into various water masses on a large scale along the vertical direction^26^. This distribution pattern was also found in our study. Based on the differences in temperature and salinity, a total of 24 samples were classified into the following groups: surface group, 200-m group, 800-m group and deep group (Supplementary Fig. S8). Furthermore, the LT2 surface seawater had a markedly high salinity compared to the salinity of the seawater from the other sites. Due to the southwest monsoon, local water masses from the SCS had an eastward flow in Luzon Strait, which weakened the invasion of the Kuroshio current^23^. Additionally, Kuroshio waters (high temperature and salinity) were not seen to cross further west than 119.5 °E and enter the SCS hinterland in the spring^22,26^. Thus, it was reasonable that the site LT2 (121.1 °E) was clearly affected by the Kuroshio waters.

Each stratification area produced homogeneous water masses harboring analogous physicochemical properties, such as temperature, salinity and nutrients^18^, which was also observed in the samples of this study (Table 1 and Supplementary Fig. S8). Water mass differences at different pelagic depths appeared to form a dispersal barrier that led to the creation of different environmental conditions and different sets of taxa^13,37^ even though there exist low-flow sinking and upwelling currents that can mix the vertical seawater body^19^. It is reported that the distance-decay relationship will be strengthened with some dispersal limitation^6^. Therefore, there are great variations between the bacterial communities found at different depths. Relative to the deeper regions, the surface water in the SCS frequently showed variable physical oceanographic processes, including monsoon-driven circulation and large-scale offshore transportation of nutrition^20,25^. These active processes in surface seawaters influenced horizontal microorganism production and movement without notable dispersal limitation. This result can further explain why no distance-decay pattern was observed along horizontal distances.

This study illustrates in detail the bacterial biogeographic patterns in the open SCS along the horizontal and vertical directions. Commonly, spatially environmental heterogeneity^34,38^ and dispersal limitation^39^ were used to interpret the observed microbial beta-diversity. The bacterial community compositions showed no detectable difference along the medium-scale horizontal geographical distances sampled in this study, showing that there is no dispersal limitation at up to ~1000 km horizontal distances in the contiguous open oceanic ecosystems. Nevertheless, bacterial community structures from different seawater layers exhibited obvious differences. Specifically, surface seawater bacteria displayed less intra-group similarities and higher inter-community diversity than deep sea bacteria. The vertical biogeographic pattern can be explained by distinct environment variables (especially DOC) and oceanographic stratification. This showed that dynamic currents and water stratification rather than simple geographic distance should be considered to interpret the bacterial biogeographical pattern in the three-dimensional contiguous seawater system. Overall, the results showed that vertical depth is the main determinant of bacterial community differentiation in the open SCS water system even though the horizontal distances were much greater than vertical depth, which will shed new lights on the understanding of microbial beta-diversity in the marine water system.

## Methods

### Location description and sample collection

Sample collection sites were located in the open SCS, far from coasts and river estuaries (Fig. 1). A total of 24 samples were collected from six sites (B3, D3, G1, F1, LT2 and X4) during a Dong Fang Hong 2 cruise in the spring of 2014. Seawater samples from each site were collected from layers at four depths, namely, 1–2 m, 200 m, 800 m and 2000–3800 m. For the purposes of this study, the 1–2 m and 2000–3800 m layers were named Surface and Deep, respectively. At each site, seawater from different depths was collected by a Sealogger conductivity-temperature-depth (CTD) oceanic rosette sampler (SBE 25, Sea-Bird Co., USA). After prefiltration through a 20-µm-pore-size nylon net membrane (Millipore Co., USA) to eliminate large particles, phytoplankton and zooplankton, five- to ten-liter seawater samples were filtered on board through 47-mm-diameter polycarbonate membranes with 0.22-µm pores (Millipore Co., USA). Filtered membranes were stored in sterile tubes (Corning Inc., USA) and kept in liquid nitrogen and then frozen at −80 °C (Thermo Scientific, USA) in the lab until nucleic acid extraction. Seawater physicochemical properties, such as temperature, salinity and water depth, were recorded by the onboard CTD Sealogger. To measure environmental parameters, water samples were filtered through 0.45-µm-pore-size cellulose acetate membranes (Millipore Co., USA). Nutrient contents (NO_2_^−^, NO_3_^−^ and PO_4_^3−^) were measured by spectrophotometric and colorimetric analyses, while dissolved organic carbon (DOC) and dissolved inorganic carbon (DIC) were calculated by a TOC analyzer (Thermo Flash 2000 Elemental Analyzer, USA).

### DNA extraction and Illumina sequencing

Total genomic DNA were extracted with a PowerWater® DNA Isolation Kit (MOBIO, USA) from the membranes. Barcoded sequencing primers 338 F (5’-ACTCCTACGGGAGGCAGCA-3’)/806 R(5’-GGACTACHVGGGTWTCTAAT-3’) were used to amplify the V3 to V4 regions of bacterial 16 S ribosomal RNA (rRNA) gene. A 20-µL PCR, containing 4 µL of 5 × FastPfu Buffer, 2 µL of 2.5 mM deoxynucleoside triphosphate (dNTP) mix, 0.8 µL of each primer (5 µM), 0.4 µL of TransStart Fastpfu DNA Polymerase (TransGen AP221–02), 1 µL of target DNA and 11 µL of double-distilled H_2_O, was performed in a PCR thermal cycler (ABI GenAmp^®^ 9700). The amplification reaction was performed in triplicate as follows: initial denaturation at 95 °C for 3 min; 27 cycles of denaturation for 30 s at 94 °C, primer annealing at 55 °C for 30 s, and chain extension for 45 s at 72 °C; and a final extension at 72 °C for 10 min. Pooled reaction mixtures were then sequenced on the Illumina Miseq PE300 sequencing platform (Majorbio Bio-Pharm Technology Co. Ltd, China) to generate large short-read libraries.

### Sequence analysis, OTU clustering and taxonomy assignment

The obtained sequences were merged and trimmed by the sequencing company. All the sequence analysis was processed by Usearch 8.0.161. The sequence reads were filtered with a maximum expected error threshold to remove unqualified portions of the sequences. After standard quality control, each trimmed sequence was approximately 450-base-pairs long. The remaining reads of the 24 seawater samples were then pooled, dereplicated, and finally assigned to each OTU using a 97% identity. Then, each sample read was mapped to representative OTU sequences of using a Perl script. For equalizing the sampling efforts of statistical comparisons, the different sample reads were matched with different assignment rates, ranging from 85% to 95%. For taxonomical assignment, the representative OTU sequences were compared with a 16 S rRNA gene database at an 80% confidence threshold by RDP Naive Bayesian rRNA Classifier (RDP Release 11.1 http://rdp.cme.msu.edu/)^40^.

### Statistical analysis

The alpha-diversity indices and Bray-Curtis similarities between samples were computed using Primer 6 (Plymouth Marine Laboratory, UK). Jaccard similarity index, bacterial community compositions and relative abundance comparisons were analyzed by R software. One-way analysis of community similarity (ANOSIM) of different layers and non-metric multidimensional scaling (NMDS) were performed in Primer 6. A standard Mantel test at each sampling layer was run in R to evaluate the correlation between horizontal geographic distance and bacterial community compositions (based on the abundances of the top 50 OTUs). Redundancy analysis (RDA) was implemented with Monte Carlo permutation tests to analyze variations in the bacterial assemblages under the constraint of environmental factors by Canoco (Version 5, Microcomputer Power). Significant environmental parameters without multicolinearity effects (variance inflation factor < 20) were obtained to explain community variability^31^. BIOENV and BVSTEP analyses were then performed in order to statistically determine correlations between community compositions and environmental variables.

### Co-occurrence analysis

Potential interaction modes were investigated through modeling the community structure networks. Positive and negative correlations in the abundances of microbial taxa were assumed to indicate co-occurrence and co-exclusion, respectively. Correlation network patterns were determined in R software with ‘Hmisc’ and ‘reshape2’ libraries and visualized in the interactive platform Gephi^41^. This platform is based on a matrix checkerboard where microbial communities interact with each other. For valid network analysis, the Spearman’s rank correlations at the phylum level were both >|0.6| and statistically significant (*P* < 0.01)^42^. At the family level, a Spearman’s correlation index of >|0.8| was calculated. To reduce network complexity, the network patterns at different taxonomic levels were constructed with >50 members across all samples^4^. In the co-occurrence networks, each node represents a single taxon (a phylum or family), and the edge between two nodes represents a positive or negative interaction between those two taxa. The degree refers to the number of edges connected to a node in a network.

The merged sequences of all 24 samples were deposited in the National Center for Biotechnology Information (NCBI) Short Read Archive database under project no. PRJNA 389159.

## Electronic supplementary material

Supplementary Information
