# Supplementary material for: Vertical and horizontal biogeographic patterns and major factors affecting bacterial communities in the open South China Sea
Source: Sci Rep. 2018 Jun 11;8:8800. doi: 10.1038/s41598-018-27191-w (PMC5995897; doi:10.1038/s41598-018-27191-w)
Supplement: Supplementary file 1 — Supplementary Information [file 41598_2018_27191_MOESM1_ESM.docx]

**Supplementary information**

**Vertical and horizontal biogeographic patterns and major factors affecting bacterial communities in the open South China Sea**

Yi Li^1^, Lin-Lin Sun^1^, Mei-Ling Sun^1^, Hai-Nan Su^1,2^, Xi-Ying Zhang^1,2^, Bin-Bin Xie^1,2^, Xiu-Lan Chen^1,2^, Yu-Zhong Zhang^1,2^, Qi-Long Qin^1,^*

^1^ Marine Biotechnology Research Center，State Key Laboratory of Microbial Technology, College of life science, Shandong University, Jinan 250100, China

^2^ Laboratory for Marine Biology and Biotechnology, Qingdao National Laboratory for Marine Science and Technology, Qingdao, China

*Corresponding author: Qi-Long Qin, [qinqilong@sdu.edu.cn](mailto:qinqilong@sdu.edu.cn)

The following tables and figures are included as Supplementary Information.

**Fig. S1.** Average diversity indexes in bacterial communities from four seawater layers. **a** Shannon Index **b** Simpson Index

**Fig. S2.** Bacterial composition distributions at phylum level across all samples. Sequences were assigned in the RDP reference database by using a 80% confidence cut-off.

**Fig. S3.** Network of co-occurrence patterns at phylum and family level from all samples. Each line represents a significant correlation between two nodes; the red lines represent positive correlations, while the green lines represent negative correlations. The size of each node is proportional to the number of connections. **a** Connections at the phylum level with Spearman’s coefficient > 0.6 or < -0.6 and *P* < 0.01, with the color indicating the topological modularity group. **b** Correlations at family level with Spearman’s coefficient > 0.8 or < -0.8 and *P* < 0.01, with each node representing a bacterial family and the color representing the phylum that the family is affiliated with.

**Fig. S4.** Correlations between bacterial community similarities at the same depth versus geographical distance. Bacterial communities were structured on Jaccard similarity. Correlations from **a** Surface, **b** 200-m, **c** 800-m and **d** Deep layers are represented as Pearson correlations.

**Fig. S5.** Bacterial community variations within each layer among four seawater layers. Community variation was built on **a** Bray–Curtis similarity and **b** Jaccard similarity, respectively.

**Fig. S6.** Variations in bacterial community similarities between different depths versus vertical geographical distance. Bacterial communities were structured on **a** Bray–Curtis similarity and **b** Jaccard similarity, respectively.

**Fig. S7.** Comparison of bacterial community similarities at different depths. The average Jaccard similarity was calculated based on the similarity values between each pair of samples from different depths.

**Fig. S8.** Relationships of temperature versus salinity of the total 24 samples.

**Table S1.** Biodiversity indexes of bacterial communities at a 0.03 distance level.

**
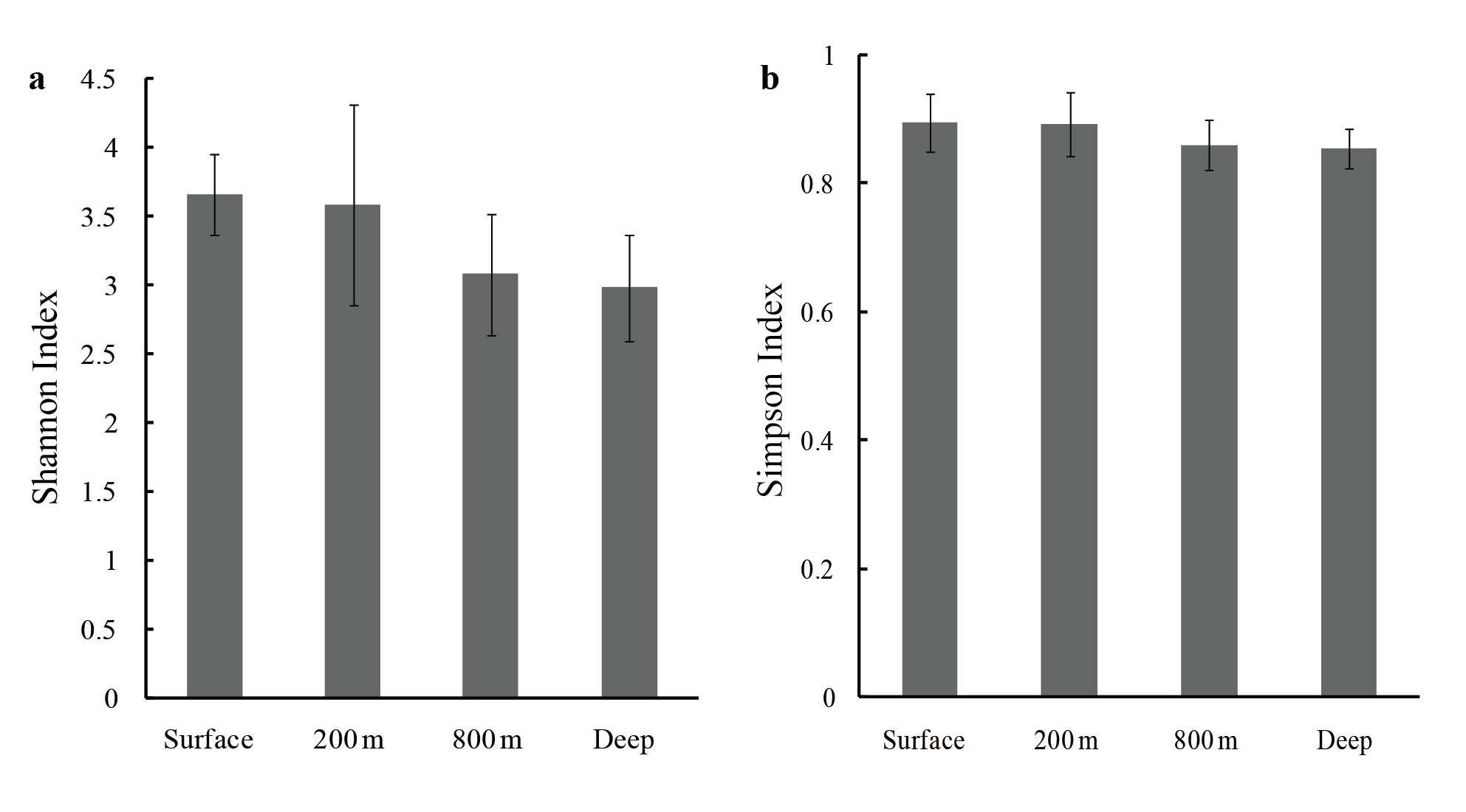
**

**Fig. S1.** Average diversity indexes in bacterial communities from four seawater layers. **a** Shannon Index **b** Simpson Index

**
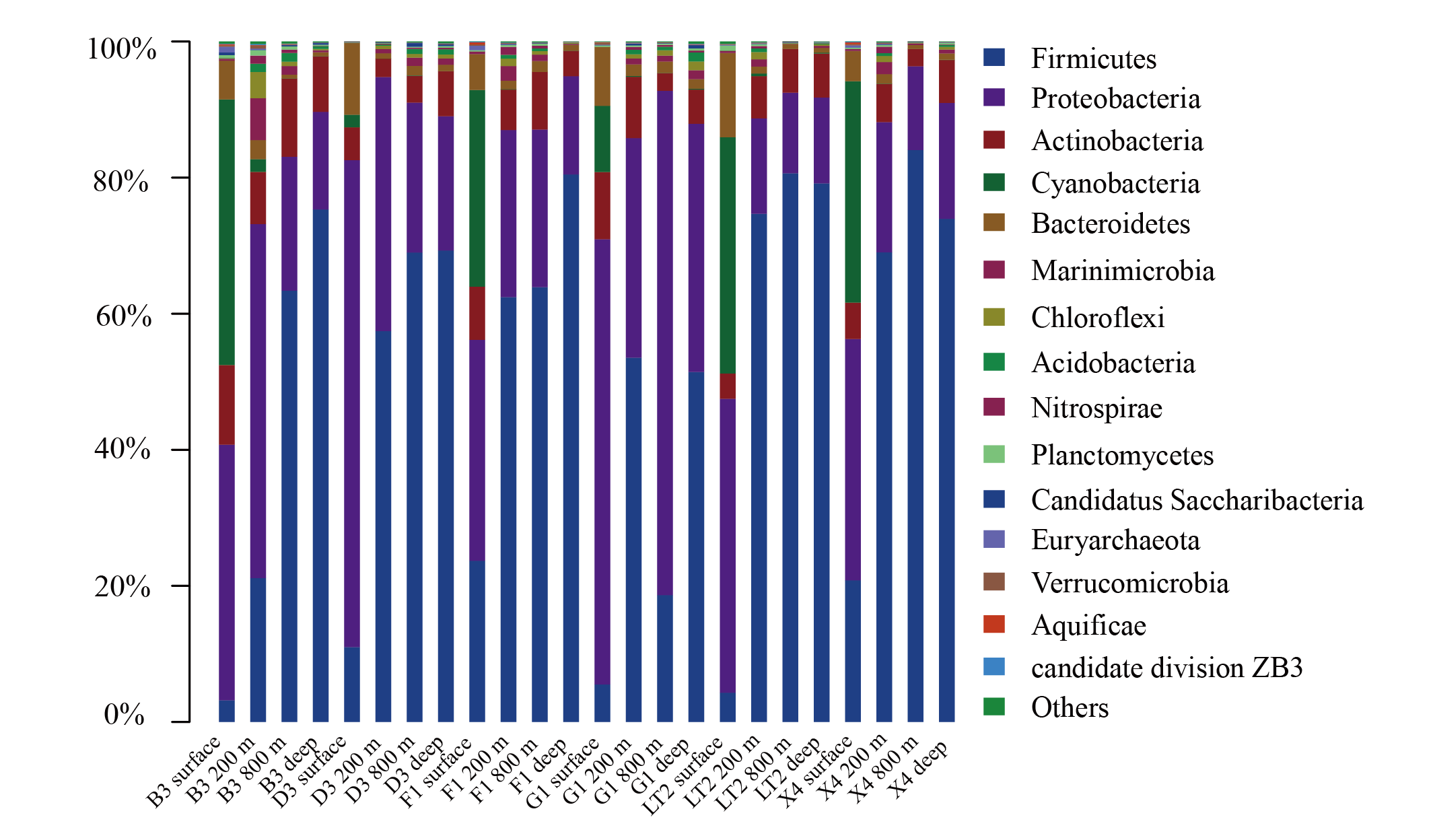
**

**Fig. S2.** Bacterial composition distributions at phylum level across all samples. Sequences were assigned in the RDP reference database by using a 80% confidence cut-off.


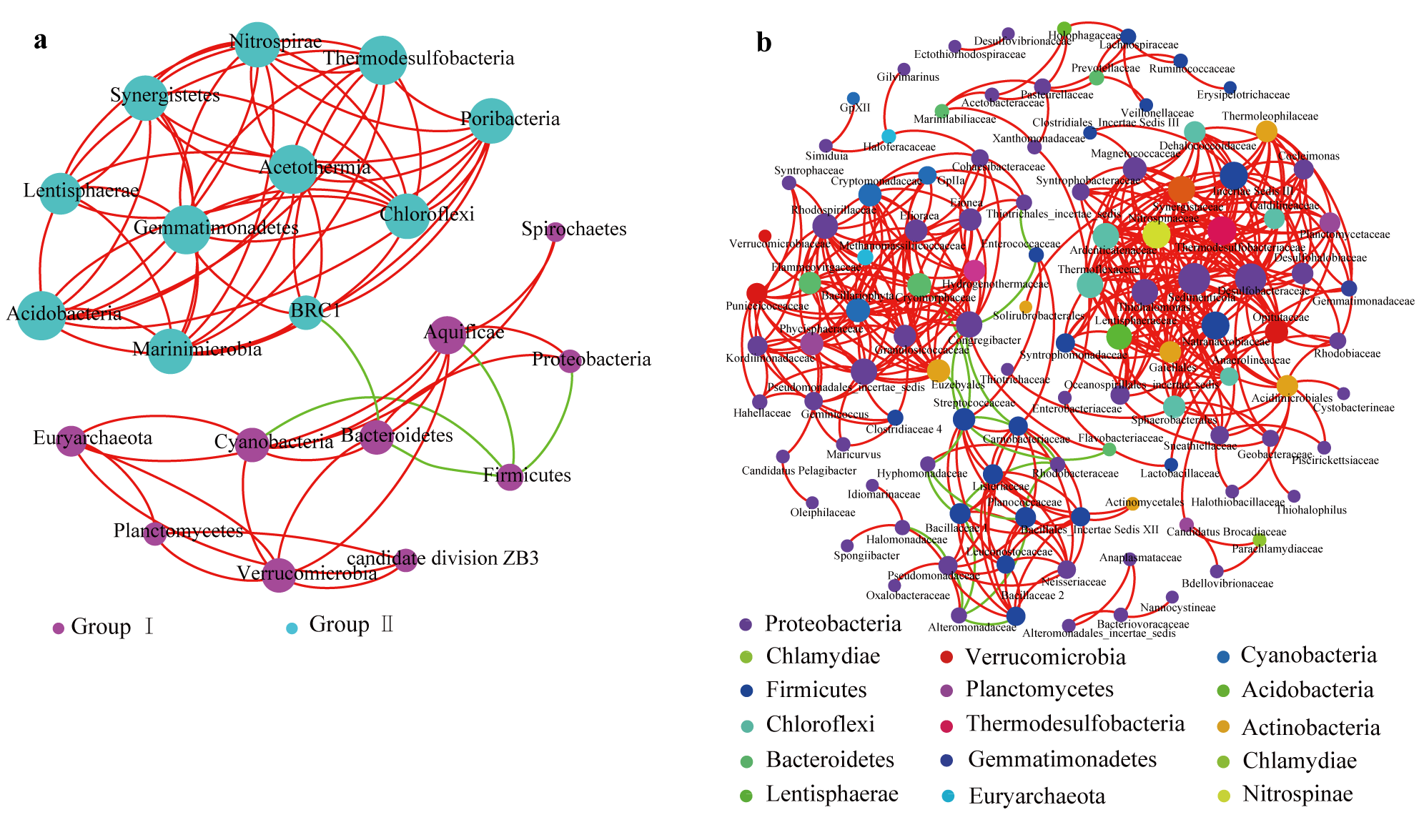


**Fig. S3.** Network of co-occurrence patterns at phylum and family level from all samples. Each line represents a significant correlation between two nodes; the red lines represent positive correlations, while the green lines represent negative correlations. The size of each node is proportional to the number of connections. **a** Connections at the phylum level with Spearman’s coefficient > 0.6 or < -0.6 and *P* < 0.01, with the color indicating the topological modularity group. **b** Correlations at family level with Spearman’s coefficient > 0.8 or < -0.8 and *P* < 0.01, with each node representing a bacterial family and the color representing the phylum that the family is affiliated with.

**
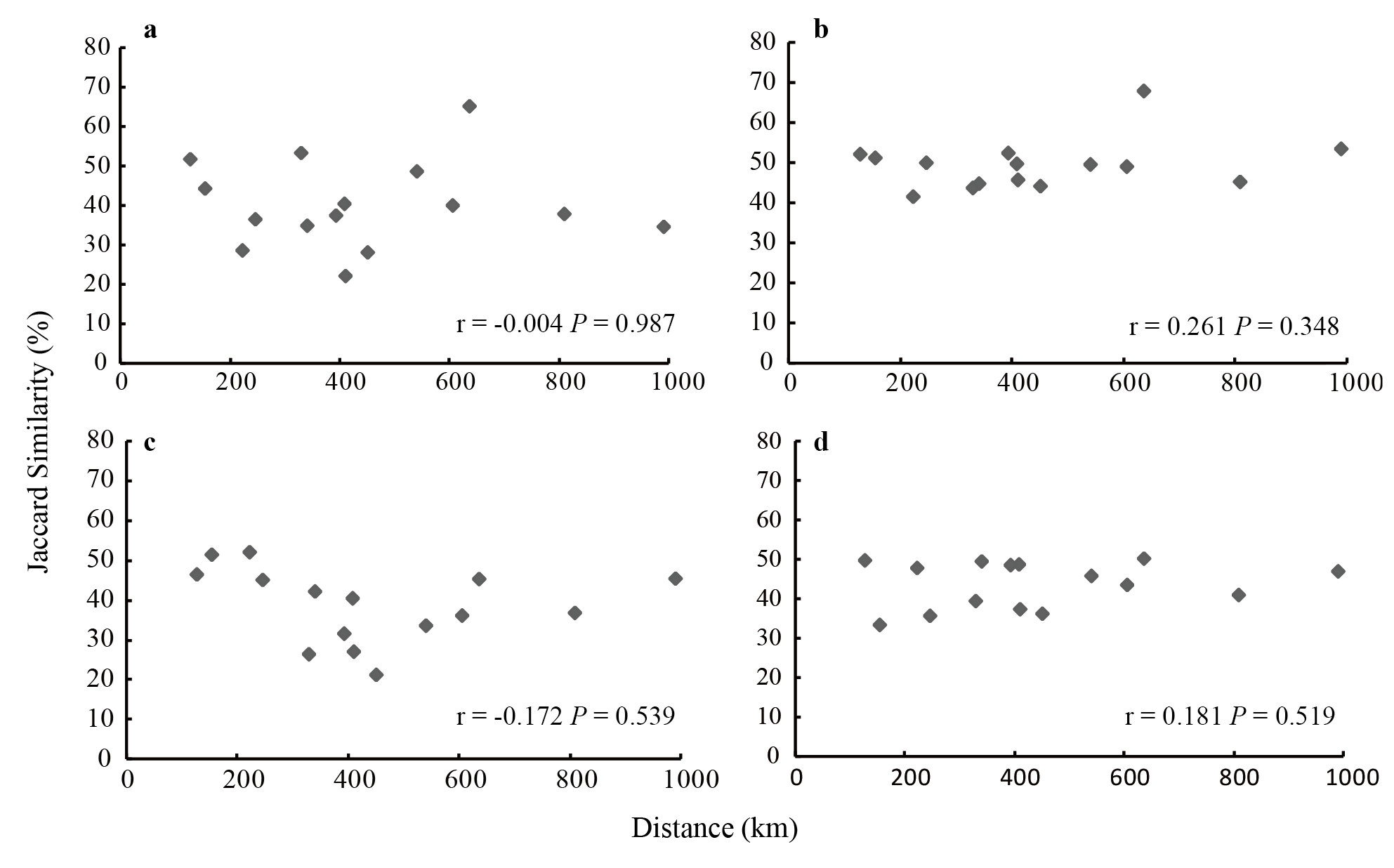
Fig. S4.** Correlations between bacterial community similarities at the same depth versus geographical distance. Bacterial communities were structured on Jaccard similarity. Correlations from **a** Surface, **b** 200-m, **c** 800-m and **d** Deep layers are represented as Pearson correlations.**
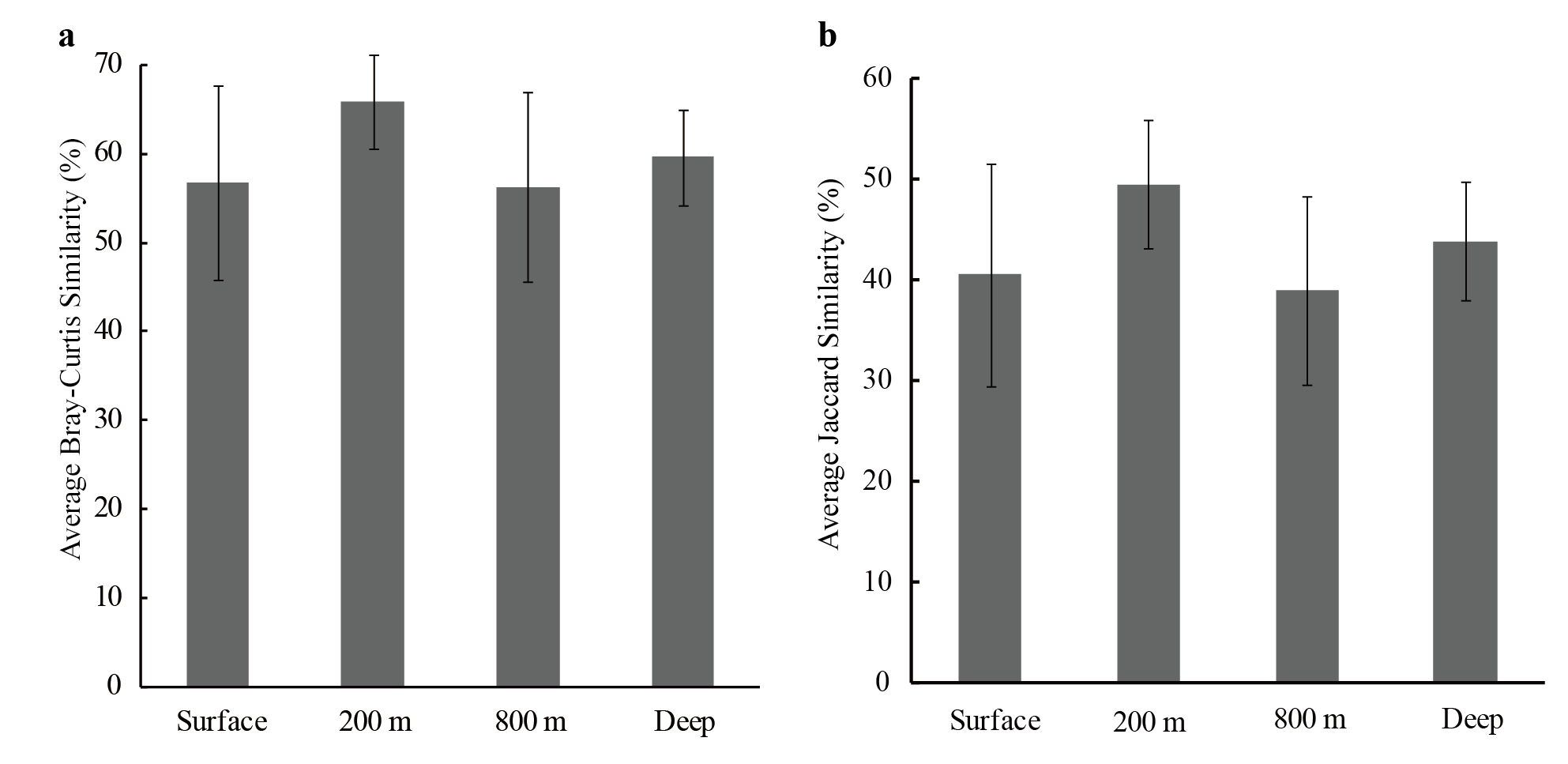
**

**Fig. S5.** Bacterial community variations within each layer among four seawater layers. Community variation was built on **a** Bray–Curtis similarity and **b** Jaccard similarity, respectively.
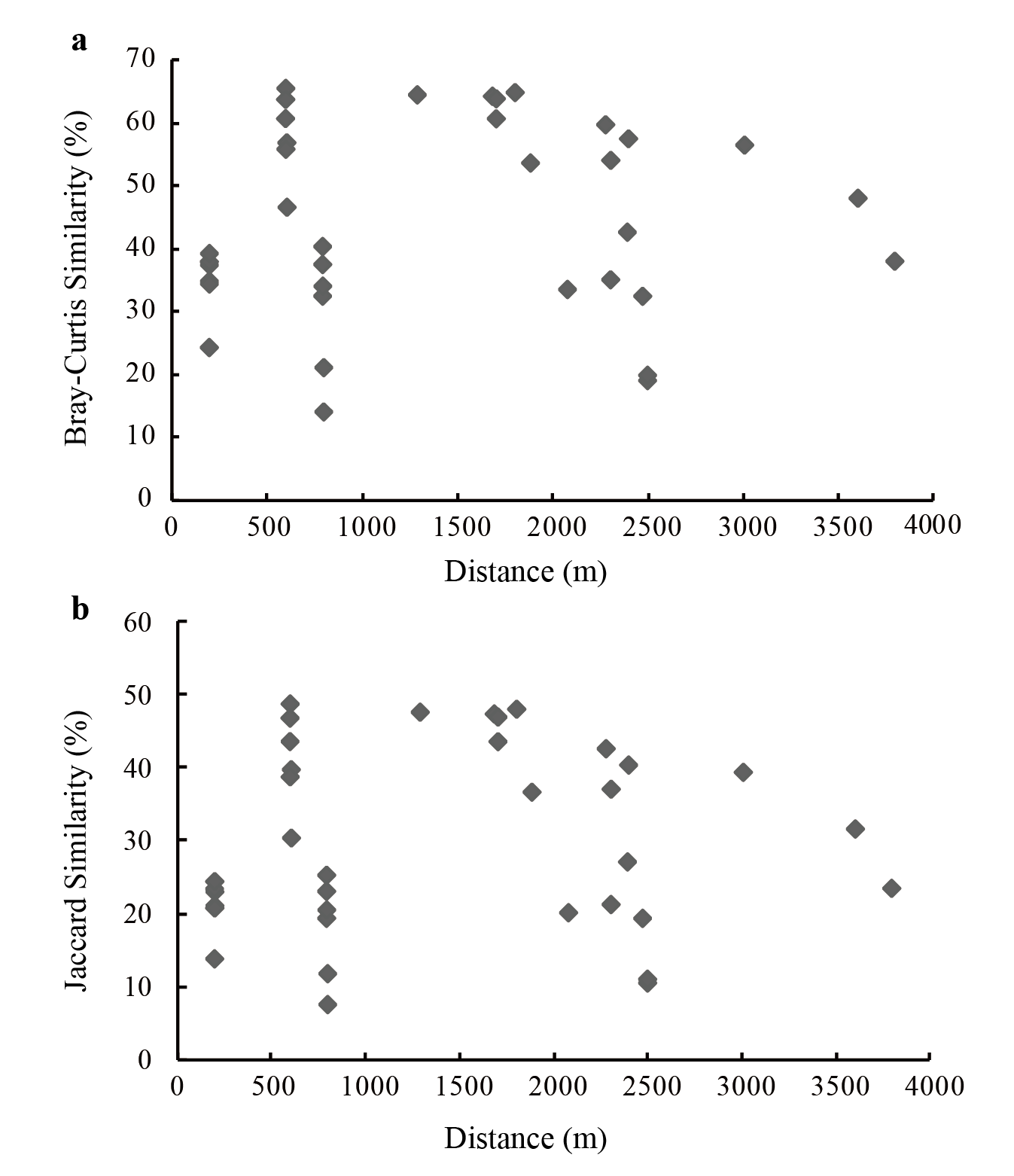


**Fig. S6.** Variations in bacterial community similarities between different depths versus vertical geographical distance. Bacterial communities were structured on **a** Bray–Curtis similarity and **b** Jaccard similarity, respectively.


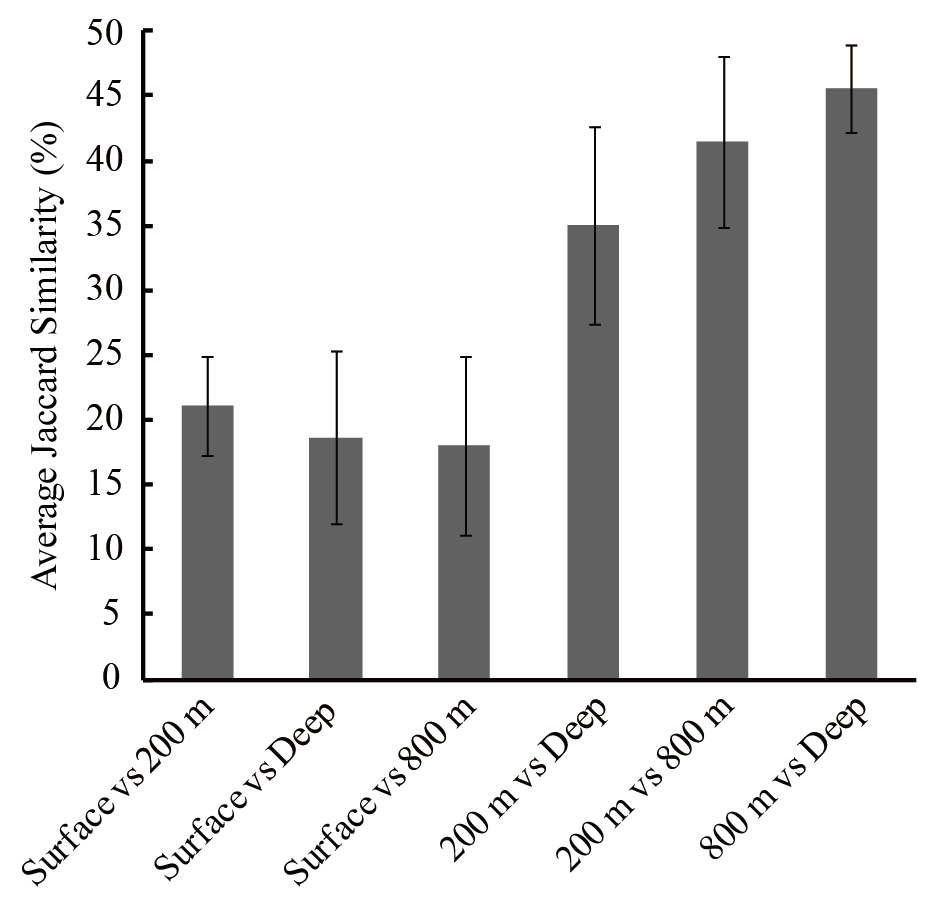


**Fig. S7.** Comparison of bacterial community similarities at different depths. The average Jaccard similarity was calculated based on the similarity values between each pair of samples from different depths.
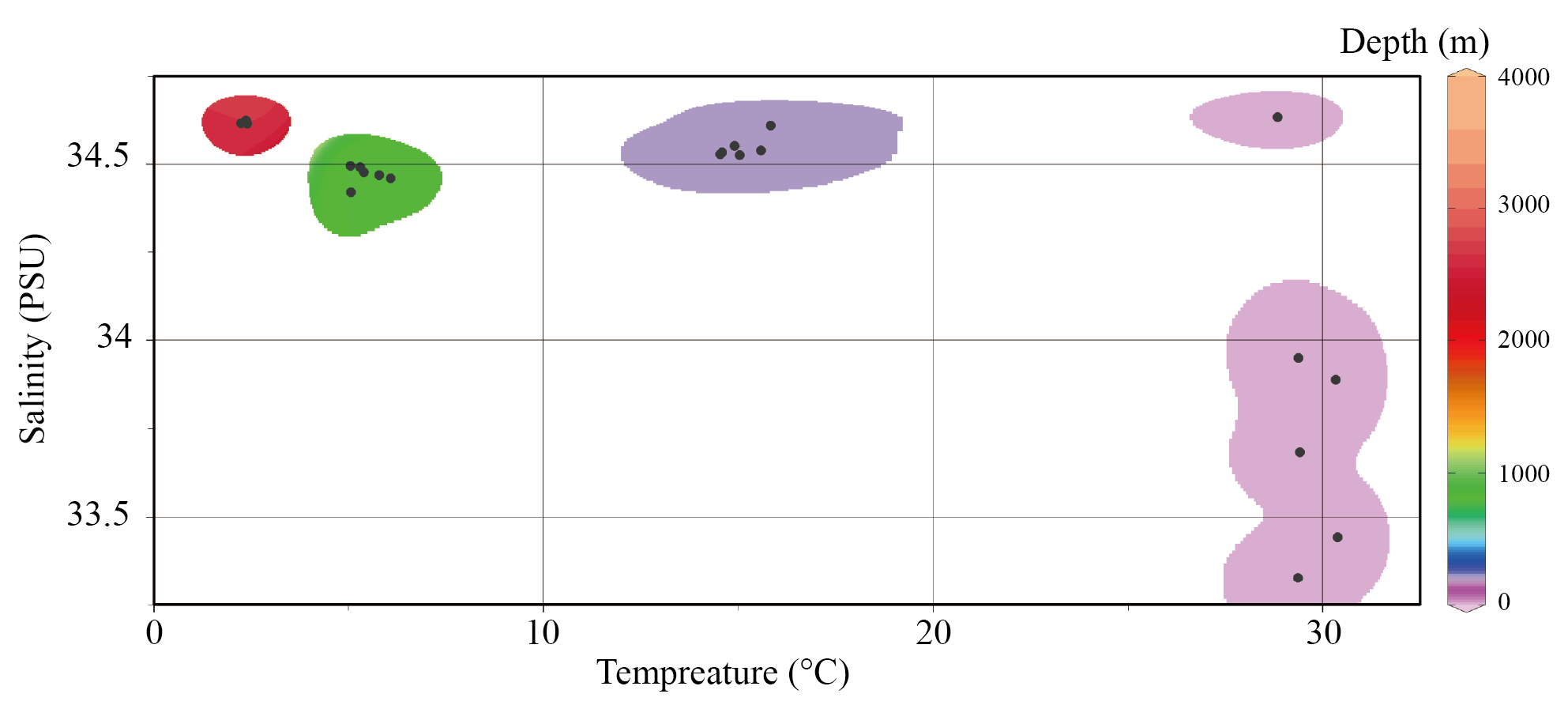
**Fig. S8.** Relationships of temperature versus salinity of the total 24 samples.

**Table S1.** Biodiversity indexes of bacterial communities at a 0.03 distance level.

| Station | Reads | OTUs | Chao 1 | Shannon | Pielou's evenness | Good's coverage |
| --- | --- | --- | --- | --- | --- | --- |
| B3 surface | 142892 | 886 | 1064 | 3.65 | 0.54 | 0.81 |
| B3 200 m | 146370 | 1310 | 1427 | 4.93 | 0.69 | 0.87 |
| B3 800 m | 105325 | 930 | 1046 | 3.30 | 0.48 | 0.82 |
| B3 deep | 137255 | 673 | 753 | 2.80 | 0.43 | 0.84 |
| D3 surface | 144141 | 712 | 837 | 3.11 | 0.47 | 0.79 |
| D3 200 m | 146479 | 903 | 1142 | 2.87 | 0.42 | 0.72 |
| D3 800 m | 109717 | 851 | 923 | 3.27 | 0.49 | 0.86 |
| D3 deep | 122385 | 807 | 900 | 3.25 | 0.49 | 0.85 |
| F1 surface | 146505 | 696 | 837 | 3.72 | 0.57 | 0.79 |
| F1 200 m | 144531 | 1007 | 1115 | 3.58 | 0.52 | 0.83 |
| F1 800 m | 131623 | 897 | 1042 | 3.43 | 0.50 | 0.79 |
| F1 deep | 121298 | 464 | 561 | 2.65 | 0.43 | 0.74 |
| G1 surface | 127741 | 802 | 906 | 3.88 | 0.58 | 0.81 |
| G1 200 m | 100321 | 1082 | 1281 | 3.71 | 0.53 | 0.80 |
| G1 800 m | 103005 | 929 | 1111 | 3.44 | 0.50 | 0.75 |
| G1 deep | 129445 | 960 | 1045 | 3.62 | 0.53 | 0.85 |
| LT2 surface | 123325 | 778 | 957 | 3.94 | 0.59 | 0.78 |
| LT2 200 m | 146948 | 931 | 1015 | 3.07 | 0.45 | 0.86 |
| LT2 800 m | 146572 | 373 | 411 | 2.55 | 0.43 | 0.81 |
| LT2 deep | 136626 | 589 | 707 | 2.64 | 0.41 | 0.72 |
| X4 surface | 122460 | 720 | 848 | 3.67 | 0.56 | 0.80 |
| X4 200 m | 133936 | 933 | 1043 | 3.35 | 0.49 | 0.83 |
| X4 800 m | 127246 | 511 | 728 | 2.47 | 0.40 | 0.64 |
| X4 deep | 131882 | 713 | 904 | 2.94 | 0.48 | 0.73 |
